# The Mediating Role of Psychological Adjustment between Peer Victimization and Social Adjustment in Adolescence

**DOI:** 10.3389/fpsyg.2016.01749

**Published:** 2016-11-10

**Authors:** Eva M. Romera, Olga Gómez-Ortiz, Rosario Ortega-Ruiz

**Affiliations:** ^1^Department of Psychology, University of CórdobaCórdoba, Spain; ^2^Department of Psychology, Social Work and Counselling, University of GreenwichLondon, UK

**Keywords:** peer victimization, bullying, social adjustment, social anxiety, negative self-esteem, social self-efficacy

## Abstract

There is extensive scientific evidence of the serious psychological and social effects that peer victimization may have on students, among them internalizing problems such as anxiety or negative self-esteem, difficulties related to low self-efficacy and lower levels of social adjustment. Although a direct relationship has been observed between victimization and these effects, it has not yet been analyzed whether there is a relationship of interdependence between all these measures of psychosocial adjustment. The aim of this study was to examine the relationship between victimization and difficulties related to social adjustment among high school students. To do so, various explanatory models were tested to determine whether psychological adjustment (negative self-esteem, social anxiety and social self-efficacy) could play a mediating role in this relationship, as suggested by other studies on academic adjustment. The sample comprised 2060 Spanish high school students (47.9% girls; mean age = 14.34). The instruments used were the scale of victimization from *European Bullying Intervention Project Questionnaire*, the negative scale from *Rosenberg Self-Esteem Scale, Social Anxiety Scale for Adolescents* and a general item about social self-efficacy, all of them self-reports. Structural equation modeling was used to analyze the data. The results confirmed the partial mediating role of negative self-esteem, social anxiety and social self-efficacy between peer victimization and social adjustment and highlight the importance of empowering victimized students to improve their self-esteem and self-efficacy and prevent social anxiety. Such problems lead to the avoidance of social interactions and social reinforcement, thus making it difficult for these students to achieve adequate social adjustment.

## Introduction

Peer victimization, which has been recognized as the most serious form of bullying, is a problem that has generated rising public concern given the negative effects such behavior has on the victims. The effects of peer victimization may even be more serious than those of other types of abuse, such as family maltreatment (Lereya et al., 2015), and may include suicidal ideation and even suicide (Díaz-Atienza et al., 2004). Although such negative consequences are basically related to the personality characteristics of the victim, given the subjectivity of the victimization process, they could also be related to the features characterizing the interpersonal dynamics surrounding the bullying phenomenon. It has been reported that victims often suffer intentional and repeated abuse by peers that exert a greater control over them, which leads them to believe they are incapable of changing the situation (Olweus, 1993; Card and Hodges, 2008). Bullying is present in all schools, affecting as many as 30% of all schoolchildren (Modecki et al., 2014), being boys and early adolescents more involved (Sánchez and Ortega, 2010) However, the percentage of boys and girls who are involved in severe and frequent bullying leading to very negative consequences is somewhat smaller but very significant, at around 10% (Hymel and Swearer, 2015).

Victimization has also been associated with intra- and interpersonal effects in the literature (see the meta-analyses of Hawker and Boulton, 2000; Reijntjes et al., 2010; and Zych et al., 2015). Although the studies in this line have focused on the direct effects of peer victimization on psychological and social adjustment, little research has been done on the interrelation between these constructs. Evidences show that psychosocial problems ocurr in victims of both sexes and in all groups of ages (Hawker and Boulton, 2000). Gender differences in adjustment is open. Although some studies find that victimization has different outcomes for boys and girls, a significant number of studies report patterns of adjustment that are the same across gender (McDougall and Vaillancourt, 2015). In early and middle adolescence, the experience of victimization predicted without differences social and psychological problems (Kumpulainen et al., 1999; Smith et al., 2004).

Social adjustment is defined as the degree to which an individual engages in competent social behavior and adapts to the immediate social context (Crick and Dodge, 1994). Research has shown that victimization experiences negatively influence social adjustment, mainly in the later stage of adolescence (Cillessen and Lansu, 2015). In this regard, it has been shown that adolescents who are victimized show low levels of social competence, acceptance and popularity (Scholte et al., 2007; De Bruyn et al., 2010), tend to be isolated and rejected (Hodges and Perry, 1999) and generally they have worse relationships with their peers (Nansel et al., 2004; Smith et al., 2004).

Indicators of psychological adjustment have also been identified as a consequences of peer victimization (McDougall and Vaillancourt, 2015). A large number of studies have shown a relationship between peer victimization and social anxiety, one of the prominent mental disorder during adolescence (Siegel et al., 2009; La Greca and Lai, 2014). Similarly, peer victimization has been found to have an impact on self-perception. Specifically, being involved in victimization is linked with low self-steem (Glüer and Lohaus, 2015; Malecki et al., 2015) and low self-efficacy (Erath et al., 2010; Kokkinos and Kipritsi, 2012) in adolescence. According to recent studies, peer relationships based on submission and low self-worth are linked to negative self-evaluations in adolescents (McDougall and Vaillancourt, 2015) and diminish their capacity to engage in satisfactory interpersonal relationships (Caprara et al., 2010).

Self-esteem is considered as the base of other self-view constructs, like self-efficacy, that influence on the style of social relationships (Kernis et al., 1989). In victimization studies that include both self-esteem and self-efficacy, it has been found that there is a positive relationship between them (Huang and Zhang, 2010; Raskauskas et al., 2015). So, it is possible that negative self-esteem favored by the victimization experience can have an inverted influence on social self-efficacy. Also, a meta-analysis of longitudinal studies suggests that low self-esteem was predictive of anxiety (Sowislo and Orh, 2013). Although these results indicate that self-esteem could influence in social self-efficacy and anxiety, all the relationships need to be studied in more depth.

It has also been recognized that social adjustment is influenced by psychological factors, among them self-esteem, social self-efficacy and anxiety. Studies on self-esteem have shown that the way in which individuals perceive themselves influences on their social, academic and emotional adjustment (DuBois et al., 1998). The relationship between social self-efficacy and social adjustment has been widely recognized (Connolly, 1989). Children who perceive that they are able to positively interact with others engage in socially acceptable behavior. However, the relationship between social self-efficacy and social adjustment depends on how the latter is measured. When social adjustment is measured in terms of maladjustment using the absence of aggressive behavior as an indicator, this relationship appears to be inverse. Thus, it has been observed that children who display aggressive behavior often feel effective when they engage in behaviors they consider socially competent. In contrast, when social maladjustment is measured as withdrawal behavior, a direct association has been found between the negative perception of social efficacy and subsequent social rejection (Connolly, 1989; Crick and Dodge, 1994). This indicates that the interpretation of the relationship between the two variables will vary, depending on the social adjustment measure used, thus casting doubt on the relationship between the two processes. For this reason, it is necessary to use instruments that are adequately suited to the definition of social adjustment. Social anxiety has also been associated with a negative assessment of one’s ability to relate effectively, as well as with low self-esteem and a lower overall academic and social self-concept (Erath et al., 2007; Delgado et al., 2013). Adolescents with social anxiety perceive themselves as being unable to cope effectively with social situations, which affects their behavior in interactive contexts (Erath et al., 2010).

Much of the research has focused on analyzing the direct effect of victimization on different indicators of social and psychological adjustment (Hawker and Boulton, 2000; Storch and Ledley, 2005; Reijntjes et al., 2010; Cillessen and Lansu, 2015), and some studies have explored the mediating relationships that could explain the effect of victimization in establishing satisfactory peer relations. Most studies have used the cognitive information processing theory to explain the influence of this demeaning experience on social adjustment. According to these studies, victims exhibit cognitive processing patterns characterized by attributing hostile and harmful intentions to peers, which causes them to fear and avoid all kinds of social situations, and ultimately, develop maladaptive social anxiety (Ziv et al., 2013). Impaired emotion regulation has been also recognized as a mediator between victimization and peer social maladjustment (Schwartz and Proctor, 2000). In any case, cognitive and emotional variables are related with social avoidance, that prevents adolescents from learning the social skills they need through peer interactions (Camodeca et al., 2015), which in turn favors negative social outcomes (Shim et al., 2013).

In this regard, it has been shown that the impact of victimization experiences on social outcomes may be mediated by psychological indicators (Wu et al., 2015). However, few studies have considered negative self-esteem, social self-efficacy and social anxiety jointly when examining the effects of peer victimization (Fredstrom et al., 2011). The relationship between these three constructs reported in the literature suggests the need to address this issue in greater depth in order to understand how psychological adjustment mediates the relationship between victimization and social adjustment of adolescents.

### The Present Study

The aim of this study is to analyze the influence of peer victimization in psychological and social adjustment. Based on literature review, three hypotheses have been put forward:

Hypothesis 1: Victimization influences social and psychological adjustment.
Hypothesis 2: Negative self-esteem, social anxiety and social self-efficacy influences social adjustment.
Hypothesis 3: Psychological adjustment plays a mediating role between victimization and social adjustment.

## Materials and Methods

### Participants

The study was carried out in accordance with the Declaration of Helsinki. The reference population used to conduct the study comprised all male and female students (368,838 in total) enrolled in compulsory secondary education (ESO; *Educación Secundaria Obligatoria* in Spanish) in the region of Andalusia (an autonomous community located in southern Spain). The sampling units were the high schools and the units of analysis were the students themselves. Prior to the data collection, informed consent was obtained from the parents to allow the children to participate in the study. In order to select the participants, random, stratified, cluster-based, probabilistic, monoetapic sampling with proportional allocation was performed. The strata were identified as geographical area (eastern or western Andalusia), type of school (public or private) and municipal population (less than 10,000 inhabitants, 10,001-100,000 inhabitants and more than 100,000 inhabitants). All the categories of the strata are relevant indices in Spain.

The study applied a 95.5% confidence level, a sampling error of 2.5%, and assumed greater variability (*p* = *q* = 0.5) (Cea D’Ancona, 1996). According to these indices, we had to select at least 1900 students. Taking into account the ratio class/students in Andalusia offered by the government (25 students per each class) and the need to select a similar number of students of all ages, we decided to select one class of each grade in each high school (100 students). So, to get a good number of students and to make up for the missing data, we needed 21 high schools.

The final sample comprised 2,060 ESO students, of which 52.1% were male and 47.9% female. The students were aged 12-19 years (*M* = 14.34; S*D* = 1.34). Of the final sample, 28.4% were in their first year of ESO, 28.4% in their second year, 22.1% in their third year, and 21.1% in their fourth year. The 95.9% of students were born in Spain.

### Instruments

The peer victimization scale was selected from *European Bullying Intervention Project Questionnaire* (EBIPQ; Ortega et al., 2016). It comprises seven Likert-type items (e.g., “Someone has hit, kicked, or pushed me”), each with five possible responses related to frequency of involvement (0 = *never*; 1 = *once or twice*; 2 = *once or twice a month*; 3 = *about once a week*; 4 = *more than once a week*). McDonald’s omega internal consistency indices yielded appropriate reliability (Ω = 0.86). Because this scale has not been validated separately, a confirmatory factor analysis was carried out to ensure its factorial structure. The model of one factor showed a good fit (χ^2^S-B = 2653.89; *p*< 0.01; NNFI = 0.90; CFI = 0.93; RMSEA = 0.07) indicating the suitability of the scale in this sample.

Negative self-esteem scale was taken from the *Rosenberg Self-Esteem Scale* (RSES; Rosenberg, 1965). It consists of four items (e.g., “At times, I think I am no good at all”) on a 4-point Likert-type scale measured according to degree of agreement. Our study has shown that the negative self-esteem scale has an acceptable internal consistency (Ω = 0.83) and a good fit of the model (χ^2^S-B = 2653.89; *p*< 0.01; NNFI = 0.90; CFI = 0.93; RMSEA = 0.07).

The *Social Anxiety Scale for Adolescents* (SAS-A; La Greca and López, 1998) was validated in Spanish adolescents by Olivares et al. (2005). The scale consists of 18 items measured on a 5-point Likert-type scale rated according to the frequency with which the respondent has experienced the symptoms described in the questionnaire (1 = *not at all*, 5 = *all the time*). The scale assesses three factors of social anxiety. The first, called *fear of negative evaluation*, measures fears, concerns or worries regarding peers’ negative evaluations and includes eight items (e.g., “I worry about what other kids think of me”); the second, called *social avoidance and distress in new situations*, measures social fears and the difficulty associated with new social situations or interactions with strangers and consists of six items (e.g., “I get nervous when I meet new kids”); while the last factor, *generalized social avoidance and distress*, measures discomfort and more general social inhibition and comprises four items (e.g., “I feel shy even with kids I know well”). In our study, the questionnaire has shown acceptable internal consistency as evaluated by McDonald’s omega (0.92 for the general scale, 0.89 for fear of negative evaluation, 0.87 for fear of new situations, and 0.84 for fear of general situations). To perform the statistical analyses, the full scale with all its correlated factors was used as a latent factor called “social anxiety”. Therefore, prior to performing the analyses, a confirmatory factor analysis of the scale was conducted following all the parameters discussed in the data analysis. The fit indices of the scale were acceptable: χ^2^S-B = 3045.58; *p*< 0.01; NNFI = 0.93; CFI = 0.94; RMSEA = 0.08.

To measure social adjustment, we used the scale *Social Adjustment* (Herrera-López et al., 2016). The social adjustment scale comprises eight items (e.g., “My classmates like me”). Responses were measured using a 7-point Likert-type scale ranging from 1 (*not at all true*) to 7 (*very true*). The scales exhibited good internal consistency in the original work and in our study (Ω = 0.90). The CFA indices of the scale in this study showed a good fit: χ^2^ = 281.04; *p*< 0.01; NNFI = 0.96; CFI = 0.94; RMSEA = 0.07.

Social self-efficacy was measured using the item “I feel I do things well (I feel successful) in relationships with my friends and classmates”. A Likert-type scale with seven degrees of agreement (1 = *strongly disagree* and 7 = *strongly agree*) was used to measure the items. Some studies use latent variables consisting of one or two items. Although this is not a widely used practice, it has led to optimal results (Bollen and Ting, 2000; Coffman and MacCallum, 2005).

### Procedure

Permission was obtained from the selected schools. Families gave written informed consent in accordance with the Declaration of Helsinki (see Supplementary Material). Ethics approval was obtained from the Coordinating Committee of Ethics of Biomedical Research of Andalusia, Spain.

The authors were responsible for the data collection. The instruments were administered to the classes as a whole in their respective classrooms without the presence of teachers in a single, 30-min session. At the beginning of the session the instructions to fill in the paper questionnaire were given. Students read all questions by themselves. The researchers were present during this time to answer any questions. Participation was entirely voluntary, confidential and anonymous. Participants were informed that they could withdraw from the study at any time.

### Statistical Analysis

Structural equation models (SEM) were developed using EQS 6.2 software. Taking into account the categorical nature of the questionnaire variables and the descriptive results of the items, where the absence of normality was evident when some variables reached values well over 0 in asymmetry and values of kurtosis greater than 2 (Bollen and Long, 1994), the least squares estimation method with robust correction was used (Bryant and Satorra, 2012). The significance of the chi-square value was tested to evaluate the goodness-of-fit of the model (values above 0.01 indicate a good fit). The value of this index is subject to other variables such as sample size (Byrne, 2014); hence, other indicators were incorporated: the comparative fit index (CFI), the non-normed fit index (NNFI; values equal to or above 0.90 indicate a good fit), the standarized root mean square residual (SRMR), the root mean square error of approximation (RMSEA; values equal to or below 0.08 indicate a good fit) and the Akaike Information Criterion (AIC; it is used to compare different models, being the lowest values optimal) (Hu and Bentler, 1999; Byrne, 2014). To ensure that the final model was appropriate for all the teenagers, the configural invariance across the age and gender groups was tested, using the same final model in girls and boys, and with students under 15 years old and with this age and older, separately.

## Results

The results of the correlation analysis between the variables are presented in **Table 1**. As can be seen, peer victimization was significantly and directly related to negative self-esteem and social anxiety, and inversely related to social self-efficacy and social adjustment. Social adjustment showed an inverse relationship with all variables, except social self-efficacy. A direct relationship was found between negative self-esteem and social anxiety. However, both variables were related inversely with social self-efficacy.

**Table 1:** Spearman’s correlations between all variables of the model.

Variables	1	2	3	4	5
Peer victimization (1)	1.00				
Social self-efficacy (2)	–0.112ˆ**	1.00
Negative self-esteem (3)	0.264ˆ**	–0.250ˆ**	1.00
Social anxiety (4)	0.302ˆ**	–0.164ˆ**	0.403ˆ**	1.00
Social adjustment (5)	–0.227ˆ**	0.479ˆ**	–0.250ˆ**	–0.285ˆ**	1.00

*^∗∗^*p*< 0.001.*

According to the first hypothesis, two models were developed. In the first model the direct relationship between victimization and social adjustment was tested. The model showed good fit (χ^2^S-B = 936.96; *p* < 0.001; NNFI = 0.94; CFI = 0.95; RMSEA = 0.06; SRMR = 0.05; AIC = 758.96) and revealed a significant direct effect of victimization on social adjustment (β = -0.33; *p* < 0.05), although the percentage of explained variance of social adjustment was low (11%). After this, the direct effect of victimization on psychological adjustment was shown with a second model whose fit was good (χ^2^S-B = 6812.87; *p* < 0.001; NNFI = 0.92; CFI = 0.93; RMSEA = 0.07; SRMR = 0.08; AIC = 6012.87). Psychological adjustment was measured by negative self-esteem, social self-efficacy, and social anxiety. The relationship between victimization, negative self-esteem (β = 0.78; *p* < 0.05), and social anxiety (β = 0.62; *p* < 0.05) was positive. However, victimization and social self-efficacy were negatively related (β = -0.35; *p* < 0.05). Victimization explained 60.6% of variance of negative self-esteem, 39% of social anxiety and 12.1% of social efficacy.

To answer hypothesis 2, a third model was tested to know if the psychological adjustment variables could influence on social adjustment. The model, which showed a good fit (χ^2^S-B = 9407.68; *p* < 0.001; NNFI = 0.96; CFI = 0.96; RMSEA = 0.04; SRMR = 0.07; AIC = 8549.68), indicated a direct relationship between negative self-esteem (β = -0.35; *p* < 0.05), social self-efficacy (β = 0.51; *p* < 0.05), social anxiety (β = -0.43; *p* < 0.05) and social adjustment. These variables explained 57% of the variance of social adjustment in total.

**FIGURE 1:**
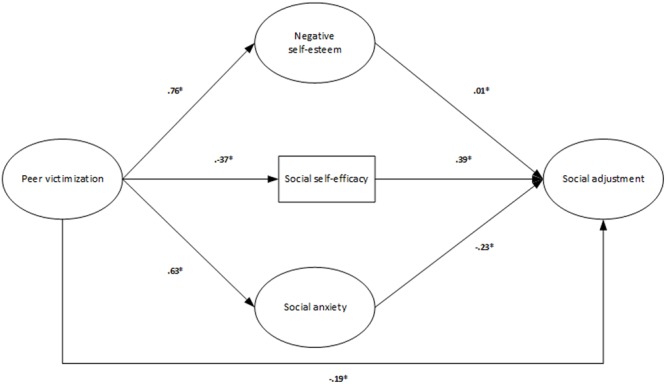
**Fourth model: direct and indirect relationships between victimization and social adjustment and direct relationship between psychological and social adjustment**.

**FIGURE 2:**
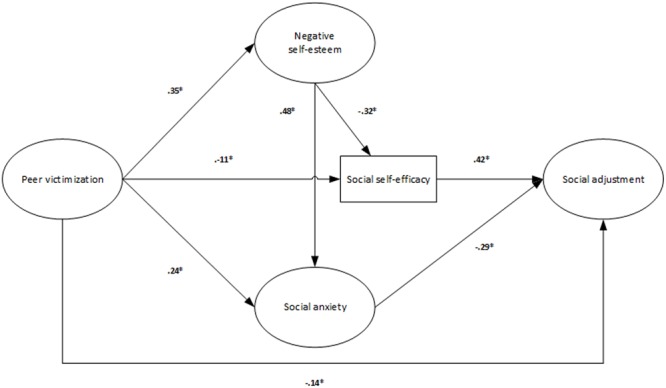
**Fifth model: direct and indirect relationships between victimization, psychological and social adjustment which place psychological adjustment as a mediator between victimization and social adjustment**.

Regarding hypothesis 3, a fourth model was built. In this model victimization was directly linked to psychological adjustment variables, and all of them were directly associated with social adjustment (see **Figure 1**). In this sense, social adjustment was directly affected by negative self-esteem (β = 0.01; *p* < 0.05), social self-efficacy (β = 0.39; *p* < 0.05), social anxiety (β = -0.23; *p* < 0.05) and victimization (β = -0.19; *p* < 0.05). Additionally, an indirect relationship was observed between victimization and social adjustment (β = -0.28; *p* < 0.05) via the relationship between the first variable and negative self-esteem (β = -0.76; *p* < 0.05), social self-efficacy (β = -0.37; *p* < 0.05) and social anxiety (β = -0.63; *p* < 0.05) (57.3, 13.4, and 40.1% of the variance of negative self-esteem, social self-efficacy, and social anxiety, respectively, was explained by its relationship with victimization). Although the model exhibited a good fit (χ^2^S-B = 8337.41; *p* < 0.001; NNFI = 0.94; CFI = 0.95; RMSEA = 0.05; SRMR = 0.06; AIC = 7025.41) and the percentage of explained variance of social adjustment was significant (38.7%), the direct effect of negative self-esteem on social adjustment was extremely weak. Given the theoretical contributions that have shown that negative self-esteem is the base of self-perceptions, conditioning self-efficacy, and it is related to psychological problems such as social anxiety, the direct relationship of this variable with social adjustment was eliminated and substituted for a direct relationship with social self-efficacy and social anxiety. An indirect relationship between negative self-esteem and social adjustment was therefore established in the model (see **Figure 2**). Although this fifth model showed a percentage of explained variance of social adjustment similar to the previous model (38.7%) its fit was better (χ^2^S-B = 7463.08; *p* < 0.001; NNFI = 0.95; CFI = 0.96; RMSEA = 0.04; SRMR = 0.06; AIC = 6153.08). In this final model, victimization (β = -0.14; *p*< 0.05), social self-efficacy (β = 0.42; *p*< 0.05) and general social anxiety (β = -0.29; *p*< 0.05) were the variables that directly influenced social adjustment. Negative self-esteem showed an indirect effect on social adjustment (β = -0.26; *p*< 0.05) through its relationship with social self-efficacy (β = -0.32; *p*< 0.05) and social anxiety (β = 0.48; *p*< 0.05), which displayed a significant and direct relationship with social adjustment. Peer victimization was also indirectly linked to social adjustment (β = -0.17; *p*< 0.05) through negative self-esteem (β = 0.35; *p*< 0.05), social self-efficacy (β = -0.11; *p*< 0.05) and social anxiety (β = 0.24; *p*< 0.05) (this relationship explained 12, 13.9, and 36.5% of the variance of negative self-esteem, social self-efficacy and social anxiety, respectively). The fit of the final model was also good in the subsamples of girls (χ^2^S-B = 4397.72; *p*< 0.001; NNFI = 0.95; CFI = 0.95; RMSEA = 0.05; SRMR = 0.06; AIC = 3089.72) and boys (χ^2^S-B = 3669.94; *p* < 0.001; NNFI = 0.96; CFI = 0.96; RMSEA = 0.04; SRMR = 0.06; AIC = 2361.94) and in those composed of the students under 15 years old (χ^2^S-B = 3627.79; *p*< 0.001; NNFI = 0.96; CFI = 0.97; RMSEA = 0.04; SRMR = 0.06; AIC = 2319.79) and with this age and older (χ^2^S-B = 4695.42; *p* < 0.001; NNFI = 0.94; CFI = 0.94; RMSEA = 0.05; SRMR = 0.07; AIC = 3387.43).

## Discussion

This study examined the relationships between peer victimization and psychological and social adjustment in a large number of adolescents. As expected, a significant association was observed.

Students who reported more experiences of victimization displayed lower levels of social adjustment. Although these results are consistent with previous studies (Nansel et al., 2004; Smith et al., 2004), most measure social adjustment as the absence of maladjustment – mainly in terms of aggression and withdrawal (Schwartz and Proctor, 2000) – or include multiple measures related to social adjustment (Scholte et al., 2007). In our study, we used a single measure that incorporates the two components of social adjustment that have been defined theoretically: socially competent behavior and peer acceptance (Crick and Dodge, 1994).

Peer victimization was found to have a positive influence on negative self-esteem and social anxiety. Social self-efficacy proved to be a psychological element with a clear influence on social adjustment that is also linked to peer victimization, as has been previously reported (Connolly, 1989). These findings support the idea that social self-efficacy as a motivating factor for achieving positive social outcomes (Ryan and Deci, 2000), is negatively influenced by victimization experiences. However, the main effect is due to feelings of negative self-esteem that arise in relationships of this type. These results are in line with studies on academic adjustment which have shown that self-efficacy influences adjustment; a relationship that is in turn mediated by negative self-esteem (Graham et al., 2006; Thijs and Verkuyten, 2008).

The mediating role of negative self-esteem between peer victimization and social adjustment is a remarkable result. Teens evaluate themselves based on the feedback they receive from their peers, which explains why negative self-esteem acts as a mediating variable between peer victimization and the other indicators of psychological adjustment (social self-efficacy and social anxiety), mainly in adolescence, a identity confusion phase where the decreased levels of self-esteem increases the vulnerability to psychological disorders (van Tuijl et al., 2014). The mediating role of negative self-esteem as a cause of other problems of psychological adjustment, such as anxiety or depression, has been recognized in recent studies (Ferro and Boyle, 2014; Jones et al., 2014). Some authors explain this relationship in terms of the attributions of the victims, who tend to blame themselves for their personal characteristics (what is known as characterological self-blame), causing them great emotional distress (Janoff-Bulman, 1979). This indicates that self-blame could be a mediating mechanism of the relationship between peer victimization and psychological maladjustment (Juvonen and Graham, 2014), thus making it of interest to develop more complex models that include social cognition variables and emotion regulation that mediate this relationship (Gómez-Ortiz et al., 2016).

The results of our study are particularly useful because they enable to evaluate a set of complex relationships that go beyond a direct analysis between variables. Peer victimization was found to have a weak effect on social adjustment and an indirect effect through psychological adjustment. Consistent with our final model, psychological adjustment acts as a partial mediator in the relationship between peer victimization and social adjustment. This approach provides insight into why victimized boys and girls exhibit problems of social adjustment. In light of these findings, it became necessary to consider self-perception and emotional distress in order to deepen our understanding of the mechanisms underlying this relationship.

## Conclusion

The present study makes important contributions to the research as it has suggested that victimized students have social adjustment problems due, in part, to problems of psychological adjustment. Our results support the partial mediating role of psychological adjustment. Social adjustment should be understood attending to the characteristics of the peer group, and our results indicate that it is also necessary to take into account the self-perceptions and emotional distress caused by victimization in order to understand the social behavior of those involved. Victimized adolescents tend to have a poor perception of themselves which makes them feel incapable of engaging in positive relationships with others and fearful of social situations. These negative effects lead them to withdraw from and forsake rewarding and satisfying relationships, thus preventing them from learning the social skills required to achieve a balanced development.

These findings have a number of practical implications that should be taken into account in the design of educational interventions. Specifically, it is necessary to improve the self-esteem of the victims and provide social opportunities in the classroom where adolescents can experience the satisfaction that comes from engaging in positive relations with others, feeling socially competent, being valued by the group and learning that not all social situations with others have to be negative, feared, or avoided. In doing so, students will improve their social adjustment with peers, which in turn becomes a protective factor against victimization experiences due to the cyclical nature of this dynamic (Juvonen and Graham, 2014).

## Limitations

Due to the cross-sectional study design, it was not possible to establish causal conclusions. However, the effects observed between the study variables coincide with longitudinal studies that recognize peer victimization as a cause of social and psychological maladjustment (Hawker and Boulton, 2000; Reijntjes et al., 2010).

Second, the study was limited due to the use of self-reports. Although the self-report method is recommended for the study of psychological adjustment variables, responses relating to victimization and psychological adjustment may be biased. Future research could include the use of sociograms that take into account the perspective of peers. Nevertheless, self-reports help to increase our knowledge of the relationship between victimization and the observed psychological effects of this phenomenon (Bouman et al., 2012).

Thirdly, the social self-efficacy measure is a limitation. This variable has been measured only through a single item, so its effects should be interpreted with caution. However, the results regarding self-efficacy as an effect of peer victimization and as an influential factor in social adjustment are in line with the scientific literature. The inclusion of social self-efficacy in the model allows us to explore this variable as a measure of psychological adjustment and draw firm conclusions about its mediating role with social adjustment.

## Future Research

A potential direction for future research could examine whether the characteristics of the peer context and friendship ties mitigate the risk of psychosocial maladjustment in victimized adolescents. Moreover, the mediating role of psychological adjustment should be explored taking into account the different forms of manifestations of victimization (physical, verbal, social), in line with future directions for research on peer victimization (Ostrov and Kamper, 2015). Other variables, such as family violence or exposure to violence should be of interest to be included in future research to assess the effect on the psychological dimensions and social adjustment. It is also necessary to consider the intensity of victimization, whose effects on psychological adjustment have been shown (van der Ploeg et al., 2015). It would also be of interest to conduct more transactional studies to determine if the psychological and social variables studied here influence victimization. The methodologies for the study of psychosocial variables focus mainly on unidirectional relations, as a cause or consequence. However, victimization has to be understood as a cyclic process, where different psychosocial variables continuously interact and influence each other (Boulton et al., 2010; Sentse et al., 2015). Establishing bidirectional relations requires the use of transactional models (Sameroff and Mackenzie, 2003). Further research needs to be done to clarify the mediating or moderating role of age and gender, in which it is considered that differences in the relation between victimization and psychosocial adjustment could depend on the frequency and multiplicity of victimization (van der Ploeg et al., 2015).

## Author Contributions

All authors listed, have made substantial, direct and intellectual contribution to the work, and approved it for publication.

## Conflict of Interest Statement

The authors declare that the research was conducted in the absence of any commercial or financial relationships that could be construed as a potential conflict of interest.
