# Supplementary material for: The Mediating Role of Psychological Adjustment between Peer Victimization and Social Adjustment in Adolescence
Source: Front Psychol. 2016 Nov 10;7:1749. doi: 10.3389/fpsyg.2016.01749 (PMC5102899; doi:10.3389/fpsyg.2016.01749)
Supplement: Supplementary file 1 [file Data_Sheet_1.pdf]

Supplementary Material

The Mediating Role of Psychological Adjustment between Peer Victimization and Social Adjustment in Adolescence

Romera, Eva M.*, Gómez-Ortiz, Olga, Ortega-Ruiz, Rosario

*** Correspondence:** eva.romera@uco.es

1 Supplementary Data

Informed Consent Form for Parent/Guardian

Dear Parent/Guardian,
From the Laboratory of Studies about Coexistence and Violence Prevention (LAECOVI, www.uco.es/laecovi) at the University of Cordoba, we are planning to undertake a survey of students in your son/daughter's school, funded by Fundación Pública Andaluza Centro de Estudios and National Plan I+D.

We would like to invite your son/daughter to participate in the survey.

The aim of this study is:

To analyse the relationship between social self-efficacy, social anxiety and peer relations to estimate risk and consequences of bullying involvement that can help to prevent it and reduce the negative effects.

The principal investigator for the study is:
Dr. Eva M. Romera, Senior Lecturer. eva.romera@uco.es from University of Cordoba. Faculty of Educational Science. Avenue San Alberto Magno St. 14004. Cordoba. Spain.

If you agree that your son/daughter agree to participate in the research:

I. *She/he will privately complete a questionnaire about bullying and peer relationships. The questionnaire is anonymous, voluntary and its information will be confidential. We estimate that it will take 30 minutes to complete the questionnaire.*

II. *To participate in this study, parents or guardians have to sign the informed consent form.*

III. *If parents or guardians and students agree to participate, the student will complete their own questionnaire individually in school. When completed he/she will deliver it to a researcher designated. If the parents or guardians and/or the student don´t want to participate in the survey, the student will be able to deliver the documents uncompleted to the researcher.*

IV. *The same questionnaire has already been completed by approximately 2000 Spanish students in 20 different schools with very different characteristics (public, private, city, town, village…etc.) without any reported adverse effects for those who participated.*

V. *The students may withdraw from the survey at any point before or during the survey without any adverse reaction from those conducting the study.*

VI. *The researcher has worked to plan this research to ensure that the greatest care will be taken to work within the school's child protection policy and procedures. All data will be anonymized when being transcribed.*

VII. *There are no benefits to participants from involvement in the research study.*

VIII. *The informed consent forms will be locked down and kept separate from the rest of the data to protect confidentiality and anonymity of data.*

IX. *If you have any questions please contact with Eva Romera (eva.romera@uco.es) and she will do her best to clarify any issues that you might have as soon as possible.*

Signature:

I have read and understood the information in this form. Therefore, I consent that my daughter/son take part in this research project.

Father/Mother/Guardian Signature: ______________________________

Name of son/daughter: ____

Date: ___________________________________
